# Impact of atrial fibrillation on the cognitive decline in Alzheimer’s disease

**DOI:** 10.1186/s13195-023-01165-1

**Published:** 2023-01-13

**Authors:** Taizen Nakase, Yasuko Tatewaki, Benjamin Thyreau, Hayato Odagiri, Naoki Tomita, Shuzo Yamamoto, Yumi Takano, Michiho Muranaka, Yasuyuki Taki

**Affiliations:** 1 Department of Aging Research and Geriatric Medicine, Institute of Development, Aging and Cancer, Tohoku University, 4-1 Seiryo Machi, Sendai, Miyagi 980-8575 Japan; 2 Smart Aging Research Center, Tohoku University, Sendai, Japan; 3 Division of Radiology, Tohoku University Hospital, Sendai, Japan

**Keywords:** Atrial fibrillation, Alzheimer’s disease, Magnetic resonance imaging, Single-photon emission tomography

## Abstract

**Background:**

Atrial fibrillation (AF) is a strong risk factor for Alzheimer’s disease (AD) independent of ischemic stroke. However, the clinicopathological impact of AF on the severity of AD has not been well elucidated. We aimed to investigate the clinical differences between dementia patients with AF and those without AF by means of imaging data.

**Methods:**

Following approval from the institutional ethics committee, patients with newly diagnosed AD or amnestic mild cognitive impairment (aMCI) were retrospectively screened (*n* = 170, 79.5 ± 7.4 years old). Cognitive function was assessed using the Mini-Mental State Examination (MMSE). Based on the MRI data, the cerebral volume, cerebral microbleeds (CMBs), periventricular white matter lesions (WMLs), and deep WMLs were evaluated. The regional cerebral blood flow (rCBF) was measured using ^123^I-IMP SPECT.

**Results:**

Of the patients, 14 (8.2%) and 156 (91.8%) had AF (AF group) and sinus rhythm (SR group), respectively. The AF group had significantly lower MMSE scores than the SR group (average [standard deviation (SD)]: 19.4 [4.4] and 22.0 [4.4], respectively; *p* = 0.0347). Cerebral volume and CMBs did not differ between the two groups. The periventricular WMLs, but not the deep WMLs, were significantly larger in the AF group than in the SR group (mean [SD] mL: 6.85 [3.78] and 4.37 [3.21], respectively; *p* = 0.0070). However, there was no significant difference in rCBF in the areas related to AD pathology between the two groups.

**Conclusion:**

AD and aMCI patients with AF showed worse cognitive decline along with larger periventricular WMLs compared to those with SR, although the reduction of rCBF was not different between patients with AF and SR. The white matter lesions may be a more important pathology than the impairment of cerebral blood flow in dementia patients with AF. A larger study is needed to confirm our findings in the future.

**Supplementary Information:**

The online version contains supplementary material available at 10.1186/s13195-023-01165-1.

## Introduction

Both atrial fibrillation (AF) and Alzheimer’s disease (AD) are medical and socioeconomic burdens on the super-aged society. AF is the main cause of cardioembolic stroke and multiple small embolisms, which could induce vascular dementia, as well as a strong risk factor for AD independent of ischemic stroke [1–4]. Regarding the underlying mechanism of AF involvement in AD pathology, several pathological conditions have been considered. A decreased total gray matter volume is associated with AF [5]. A smaller hippocampal volume has been observed in patients with AF than in controls [6]. Decreased cerebral blood flow (CBF) and perfusion of brain tissue were observed in patients with AF by the analysis of phase-contrast MRI data [7]. AF patients with heart failure present with decreased CBF velocity and worse cognitive impairment [8]. Furthermore, AF has been reported to induce an inflammatory response [9, 10], which is related to neuronal damage and dementia. However, several negative reports have also been published. AD patients with AF or heart failure presented with older ages of dementia onset and death than AD patients without heart disease [11]. AD patients with cerebrovascular diseases (CVD) also showed older ages at death than AD patients without CVD [12]. They observed that neuritic plaque density did not differ between AD patients with and without CVD. The involvement of AF in AD pathology is still controversial. In this study, we aimed to determine whether AF influences the severity of dementia and investigate any pathological difference based on brain imaging studies between dementia patients with and without AF.

## Methods

### Patients

All procedures in this study were approved by the independent ethics committee of the Tohoku University School of Medicine (#2021-1-385). Written informed consent was obtained from all participants.

**Fig. 1:**
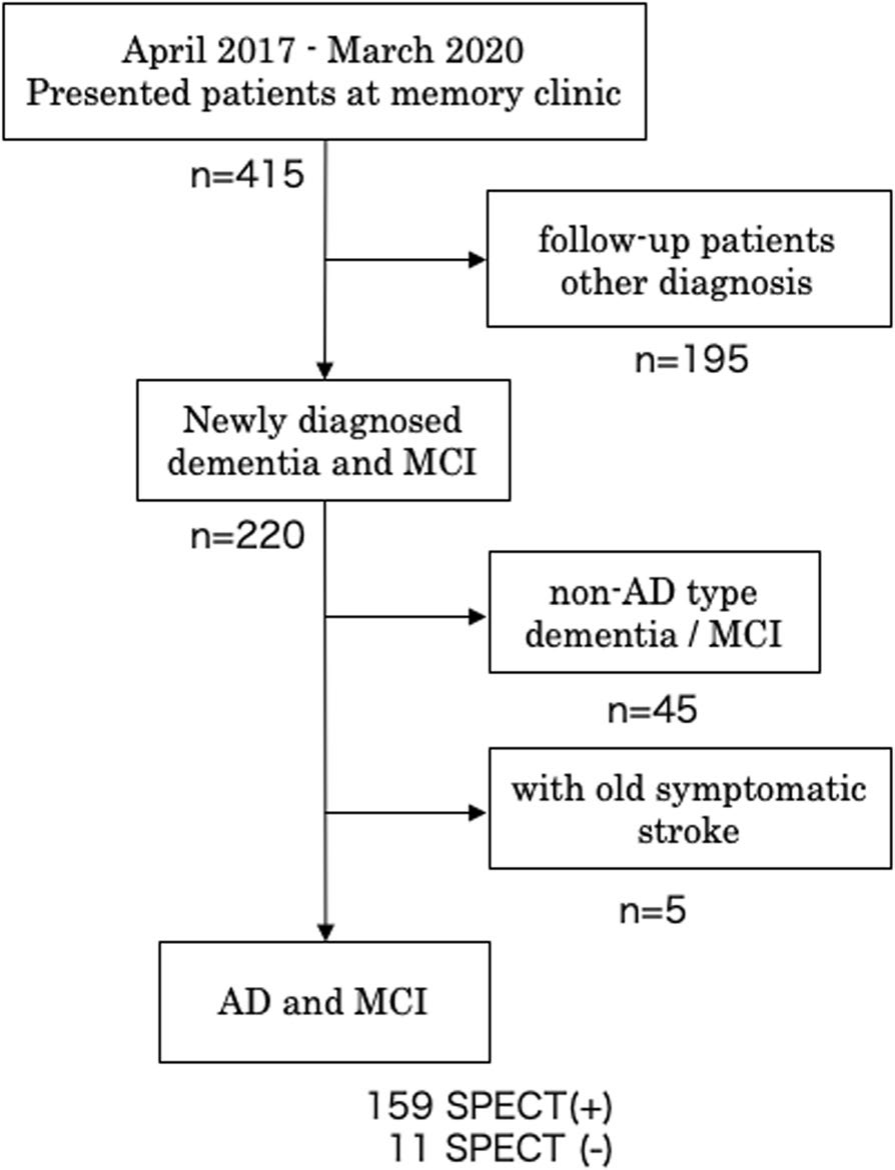
Patient selection algorithm. AD, Alzheimer’s disease; MCI, mild cognitive impairment

We screened consecutive new patients who visited our memory clinic between April 2017 and March 2020 (*n* = 220). Patients diagnosed with AD or amnestic mild cognitive impairment (aMCI) by registered neurologists or gerontologists following the guidelines [13, 14] were enrolled in this study (*n* = 170). The number and average [SD] age of patients with AD and aMCI were 115 (80.5 [6.8] years old) and 55 (77.4 [8.0] years old), respectively. The patient selection algorithm is shown in Fig. 1. The exclusion criteria were (1) patients with a diagnosis other than dementia, (2) patients with non-AD type dementia and MCI, and (3) patients with old symptomatic stroke. All the patients underwent brain magnetic resonance imaging (MRI: Intera Achieva 3.0T Quasar Dual, Philips, Amsterdam, The Netherlands) and neuropsychological tests. The diagnosis of chronic AF was confirmed based on the patient’s clinical records. All enrolled patients were classified into a comorbid AF group (AF group) and a sinus rhythm group (SR group). Personal educational background, medical history, comorbidities (such as hypertension, hyperlipidemia, and diabetes mellitus), and prescribed medicines were extracted from the medical records at the first visit. Laboratory data were obtained from the blood examination at the first visit. Cognitive impairment was assessed using the Japanese version of the Mini-Mental State Examination (MMSE-J) performed within 1 month following the first visit.

### Imaging data analysis

For obtaining cerebral volume, brain MRI T1-weighted images (T1-WI) were processed using an automatic in-house pipeline based on the Statistical Parametric Mapping (SPM)-Lesion Segmentation Tool toolbox (https://www.applied-statistics.de/lst.html). The cerebral areas of the 2D images were measured and summed for all areas to obtain the whole cerebral volume and presented in units of milliliters. Old cerebral ischemic lesions, including lacunar infarction, were assessed on T2-WI. Cerebral microbleeds (CMBs) were counted on T2*-WI in the brainstem, cerebellum, basal ganglia, and subcortical white matter, separately. MRI fluid-attenuated inversion recovery (FLAIR) images were used to analyze periventricular (PV) white matter lesions (WMLs) (PVWMLs) and deep WMLs (DWMLs). To obtain the WML volume (presented in units of milliliters), high-intensity lesions on the FLAIR images were measured using the same computer software as described above. The periventricular region was defined as the region attached to the ventricles, and a subcortical region was defined as a ribbon mask of the below gray matter; the rest was labeled as deep white matter. From the lesion segmentation and region definition, we calculated the total lesion volume for each brain region for all acquisitions for every participant. Magnetic resonance angiography (MRA) was used to analyze the vessel stenosis in the major brain arteries.

Regional cerebral blood flow (rCBF) was measured using N-isopropyl-p-^123^I-iodoamphetamine single-photon emission tomography (SPECT) data. The relative reduction of rCBF in a specific area of the brain was calculated using stereotactic extraction estimation (SEE) analysis, which was implemented in the SPECT system (Symbia-E, Siemens Healthcare Inc.). The regions of interest were the superior frontal gyrus, medial frontal gyrus, precuneus, thalamus, parahippocampal gyrus, and posterior cingulate gyrus.

### Statistical analysis

Data are presented as numbers and percentages or as mean ± standard deviation (SD). The comorbid atherosclerotic risk factors in the AF and SR groups were compared as percentages using the chi-squared test. As the age and MMSE-J scores did not follow a normal distribution, the Wilcoxon test was used to compare the differences between the AF and SR groups. Laboratory data of the two groups were compared using a *t*-test. For the multivariate analysis, confounding factors which showed a difference with *p* < 0.05 in the bivariate analysis were adopted. Moreover, as confounding factors might not be in normal distribution and contain both continuous variables and nominal qualitative variables, Spearman’s rank correlation coefficient test and stepwise regression analysis were used for analyzing the association with the lowering of the MMSE-J score. The differences in SEE data in each brain area were compared using one-way analysis of variance (ANOVA) and Tukey’s test.

The JMP Pro15 software was used for the analysis. *p* < 0.05 was defined as significance.

## Results

### Patient clinical characteristics

There were 14 (8.2%) patients in the AF group and 156 (91.8%) in the SR group. The clinical characteristics of all the patients are shown in Table 1. The percentage of AD patients in the AF group was significantly higher than that in the SR group (*p* = 0.0382: 92.9% and 65.4%, respectively). Patients in the AF group had significantly lower MMSE-J scores (average [SD]) than those in the SR group (19.4 [3.1] vs 22.0 [4.4]: *p* = 0.0347, respectively). The average [SD] age at the first visit was not significantly different between the AF and SR groups (81.6 [4.1] and 79.3 [7.6] years old, respectively; *p* = 0.2575). The frequencies of hypertension and dyslipidemia were not significantly different between the AF and SR groups, but the frequency of diabetes mellitus was significantly higher in the AF group than in the SR group (50.0% and 23.7%, respectively; *p* = 0.0315). The average [SD] HbA1c was not significantly different between the AF and SR groups (6.4 [0.78]% and 6.1 [0.87]%, respectively; *p* = 0.2211). The prevalence of dysthyroidism (hyper- or hypothyroidism assessed by laboratory data) did not differ between the two groups. The cardiothoracic ratio (CTR) measured on the chest X-ray image was significantly higher for the AF group than for the SR group (mean [SD]%, 57.6 [5.9] and 50.0 [5.6], respectively; *p* < 0.0001). There were no significant differences between the systolic and diastolic blood pressures of the two groups. Renal function and hemoglobin concentration were not different between the two groups. The inflammation-related data, i.e., WBC, CRP, and homocysteine, were not significantly different between the two groups. Antithrombotic therapy was administered to 88.2% of the patients with AF. However, the doses of three patients were not optimal; that is, three patients taking warfarin showed a prothrombin time international normalized ratio (PT-INR) of < 1.6.

**Table 1:** Patient characteristics

	Total	AF	SR	***p***
**n**	170	14	156	
**m/f**	65/105	5/9	60/96	0.8394
**AD/aMCI (n, AD%)**	115/55 (67.6%)	13/1 (92.9%)	102/54 (65.4%)	0.0382
**MMSE-J score (ave. ± SD)**	21.8 ± 4.3	22.0 ± 4.4	19.4 ± 3.1	0.0347
**Age (ave. ± SD, y.o.)**	79.5 ± 7.4	81.6 ± 4.1	79.3 ± 7.6	0.2575
**Hypertension (%)**	65.9	71.4	65.4	0.6477
**Dyslipidemia (%)**	37.7	28.6	38.5	0.4644
**Diabetes mellitus (%)**	25.9	50.0	23.7	0.0315
**Dysthyroidism (%)**	4.7	0.0	5.2	0.3838
**CTR (ave. ± SD, %)**	50.7 ± 5.8	57.6 ± 5.9	50.0 ± 5.6	0.0347
**Systolic BP (ave. ± SD, mmHg)**	139.3 ± 22.1	146.6 ± 35.5	137.1 ± 21.6	0.1384
**Diastolic BP (ave. ± SD, mmHg)**	74.0 ± 13.2	74.9 ± 18.8	73.2 ± 12.8	0.6361
**eGFR (ave. ± SD, ml/min/1.73 m**^**2**^**)**	68.4 ± 19.7	65.4 ± 17.8	68.3 ± 20.0	0.5965
**Hemoglobin (ave. ± SD, mg/dL)**	12.8 ± 1.8	13.3 ± 1.5	12.8 ± 1.8	0.3171
**WBC (ave. ± SD, /μL)**	5918 ± 1517.2	6079 ± 1911.6	5801 ± 1541.4	0.5284
**CRP (ave. ± SD, mg/dL)**	0.181 ± 0.409	0.229 ± 0.388	0.175 ± 0.407	0.6453
**Homocysteine (ave. ± SD, mg/dL)**	14.1 ± 10.0	13.4 ± 5.8	14.1 ± 10.5	0.8092
**Vitamin B12 (ave. ± SD, mg/dL)**	620.1 ± 810.4	785.2 ± 695.9	605.6 ± 806.5	0.4380
**Folic acid (ave. ± SD, mg/dL)**	9.8 ± 5.3	10.3 ± 6.2	9.7 ± 5.1	0.6952

*AD* Alzheimer’s disease, *aMCI* amnestic mild cognitive impairment, *ave.* average, *SD* standard deviation, *y.o.* years old, *MMSE-J* Japanese version of Mini-Mental State Examination, *CTR* cardio-thoracic ratio, *BP* blood pressure, *eGFR* estimated glomerular filtration rate, *WBC* white blood cell, *CRP* C-reactive protein

### Affected white matter lesions by AF

**Fig. 2:**
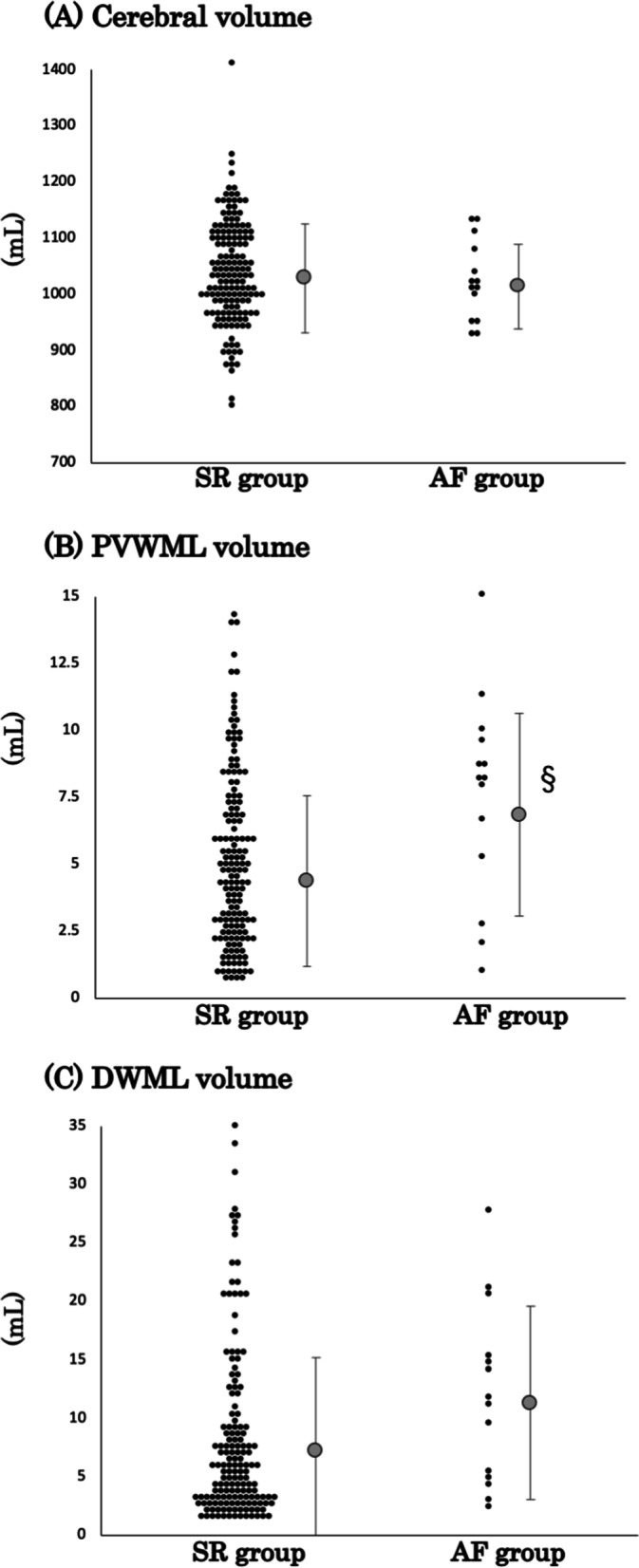
Scatter graphs of cerebral volume (**A**), PVWML volume (**B**), and DWML volume (**C**). There was no difference in the distributions of cerebral volume in the AF and SR groups. The PVWML volume in the AF group was significantly increased (^§^*p* = 0.0070). Regarding the distribution of DWML volume, the AF group had a greater volume than the SR group, although the difference was not significant (*p* = 0.0699). The *Y*-axis represents the volume in milliliters. The gray circles and bars on the right side of the scatter plots indicate the averages and standard deviations, respectively. PVWML, periventricular white matter lesion; DWML, deep white matter lesion

There was no significant difference in cerebral volume between the AF and SR groups (average [SD] mL: 1014 [74.9] and 1029 [97.0], respectively; *p* = 0.5741) (Fig. 2A). The volume of PVWML was significantly greater for the AF group than for the SR group (mean [SD] mL: 6.85 [3.78] vs 4.37 [3.21], respectively; *p* = 0.0070) (Fig. 2B). The volume of DWML was relatively greater in the AF group than in the SR group (mean [SD] mL: 11.34 [8.25] vs 7.26 [8.00], respectively; *p* = 0.0699) (Fig. 2C). Even though patients with AD and aMCI were separately analyzed, these differences showed the same results (Fig. S1). The existence of any old ischemic lesions in the cerebrum was observed in 15.4%, 8.8%, and 9.3% of AD patients in the AF group, AD patients in the SR group, and aMCI patients in the SR group, respectively. These percentages were not significantly different (Additional file 2: Table S1). The numbers of CMBs in the subcortical and the deep white matter regions showed no significant difference between the AF and SR groups (average [SD]; 1.2 [4.3] vs 2.5 [7.9], *p* = 0.4463, and 0.2 [0.4] vs 0.3 [1.5], *p* = 0.5201, respectively). The trend was preserved even if patients were separated into AD and aMCI (Additional file 3: Table S2). According to the MRA findings, no patient showed significant stenosis in the main arteries of the brain (data not shown).

### Multivariate analysis

Confounding factors which presented the significant difference between the AF and SR groups by the bivariate analysis were adopted for the multivariate analysis, i.e., MMSE-J score, diabetes mellitus, CTR, and PVWML. CTR and PVWML still showed a significant correlation with AF (correlation coefficient [CC] = 0.231, *p* = 0.003, and CC = 0.365, *p* < 0.001, respectively). However, the MMSE-J score did not present a significant correlation with AF (CC = − 0.122, *p* = 0.120). Diabetes mellitus also showed no significant correlation (CC = 0.147, *p* = 0.062). Moreover, patients with AD were separately analyzed, significant correlation with AF was also observed in CTR and PVWML (CC = 0.348, *p* < 0.001, and CC = 0.214, *p* = 0.022, respectively). MMSE-J score and diabetes mellitus did not show a significant correlation with AF (CC = − 0.085, *p* = 0.367, and CC = 0.122, *p* = 0.194, respectively). When the involvement of each factor in the lowering of the MMSE-J score was assessed by Spearman’s rank correlation coefficient test, only the presence of AF showed a significant contribution (Table 2). While the stepwise regression analysis was adopted with minimal BIC rule, AF also showed a significant correlation with the lowering of the MMSE-J score (Additional file 4: Table S3).

**Table 2:** Spearman’s rank correlation coefficient test for the lowering of MMSE-J score

	MMSE-J score	*p*
**Atrial fibrillation**	− 0.1893	0.0134
**Diabetes mellitus**	− 0.1183	0.1245
**CTR**	− 0.1481	0.0540
**PVWML**	− 0.0690	0.3711

*MMSE-J* Mini-Mental State Examination Japanese version, *CTR* cardiothoracic ratio, *PVWML* periventricular white matter lesion

### Retained cerebral blood flow in AF

rCBF was calculated using the SEE analysis of SPECT data in a specific area of the brain, which is expected to be related to AD pathology (Table 3). Both the AF and SR groups showed decreased rCBF in these areas. However, there were no significant differences in the data between the AF and SR groups. Instead, the rCBF reduction was relatively remarkable in the AD-related areas for the SR group than for the AF group. These trends became more remarkable, when patients with AD and aMCI were separately analyzed (Additional file 5: Table S4).

**Table 3:** Stereotactic extraction estimation analysis for SPECT

	AF	SR	*p*
n	12	147	
**Superior frontal gyrus (L/R, ave. ± SD)**	71.6 ± 25.3/72.9 ± 28.0	64.0 ± 27.6/62.6 ± 28.8	0.3595/0.2350
**Medial frontal gyrus (L/R, ave. ± SD)**	75.7 ± 18.8/75.9 ± 21.9	66.7 ± 25.1/66.5 ± 26.1	0.2300/0.2304
**Precuneus (L/R, ave. ± SD)**	71.3 ± 32.4/74.8 ± 33.4	66.3 ± 31.3/68.3 ± 31.7	0.5970/0.4989
**Thalamus (L/R, ave. ± SD)**	86.9 ± 24.5/89.0 ± 26.9	70.0 ± 34.1/80.9 ± 29.3	0.0958/0.3573
**Parahippocampal gyrus (L/R, ave. ± SD)**	82.1 ± 16.4/85.0 ± 16.1	73.5 ± 29.9/74.5 ± 29.2	0.3273/0.2194
**Posterior cingulate gyrus (L/R, ave. ± SD)**	83.5 ± 26.8/87.2 ± 23.9	78.4 ± 25.6/74.9 ± 26.6	0.5070/0.1223

*L* left, *R* right, *ave.* average, *SD* standard deviation

## Discussion

Our results demonstrated that patients with AD and amnestic MCI with AF have more severe cognitive impairment than those with SR. Periventricular white matter pathology may contribute to cognitive decline in addition to AD-related lesions in these patients.

Although AF is a major cause of embolic stroke, a recent meta-analysis reported that AF presented with a higher risk of dementia independent of ischemic stroke incidence (observation of an average of 6.8 years among 60,000 cases; HR 1.28) [4]. Regarding the pathological relationship between AF and AD, it was reported that patients with AF showed significantly impaired cognitive function and lower hippocampal volume than controls with adjustment for covariates [6]. Another study, which used data from the Atherosclerosis Risks in Communities (ARIC) study, reported that AF was associated with worsening of cortical atrophy by longitudinal observation; however, there was no association between AF and the volumes of the whole brain and WMLs by cross-sectional analysis [15]. Moreover, some studies mentioned that there was no relation between AD pathology and the presence of AF [11, 12]. It can be said that the involvement of AF in the pathogenesis of AD has various aspects. In this study, a more severe cognitive decline was observed in patients with AF than in those with SR. Since clinical characteristics were not different between patients with AF and SR, our findings may support the idea that AF influences the worsening of cognitive impairment in patients with dementia. We also found that the cerebral volume was not significantly different between patients with AF and those with SR, although patients with AF showed significantly higher PVWML volumes and relatively high DWML volumes than those with SR. According to the multivariate analysis, AF significantly correlated with PVWML, and AF significantly influenced the severity of cognitive impairment. Although we did not find a relation between PVWML and MMSE-J score, it will be noteworthy to investigate the involvement of PVWML in declining cognitive function among patients with AF. Meanwhile, the percentage of diabetes mellitus was significantly high in the AF group; however, comorbid ischemic lesions were not different between the AF and SR groups, and multivariate analysis revealed that diabetes mellitus did not critically affect cognitive decline. Of note, diabetes mellitus has been reported as a risk of AD [16], and we need to consider its influence on the severity of cognitive decline and PVWML in a larger study.

Moreover, we observed that the reduction in regional cerebral perfusion in areas related to AD pathology was generally less in patients with AF than in those with SR, although the severity of cognitive impairment was remarkable in patients with AF. It can be said that there is a possibility that AF may modify cognitive decline along with AD pathology. Recent studies have reported that AF can induce not only cerebral hypoperfusion but also inflammatory response [9, 10]. Patients with AF have been reported to show increased serum concentrations of TNF-α, MCP-1, IL-8, and NT-pro BNP [10]. Ablation therapy, which is a treatment for maintaining cardiac sinus rhythm, can reduce C-reactive protein concentrations [17]. We also reported that a cognitive function was recovered 6 months after ablation along with an improvement of regional perfusion in the posterior cingulate gyrus [18]. As inflammation is one of the devastating factors of vascular endothelia, we need to take into account the effect of the inflammatory response on the pathogenesis of PVWMLs in AF patients. Notably, it was reported that severe PVWMLs were observed twice more frequently than severe subcortical WMLs in patients with AF [19]. PVWML was associated with worse perivascular ependymal damage and fewer ischemic insults than DWML [20, 21]. Investigating the difference in the distribution of WMLs may help understand the involvement of AF in AD pathology.

In this study, we collectively investigated the influence of AF on cognitive decline in both AD patients and amnestic MCI patients, since amnestic MCI can be considered on the same spectrum as AD pathology [22]. In fact, even though patients were separately analyzed in AD and aMCI, the results showed the same trends.

### Limitations

This study had several limitations. First, the enrolled patients, particularly those with AF, were few. In this regard, the statistical power of this study was 0.932 by calculation using current data obtained from the AF group (*α* = 0.05, SD = 3, *n* = 14, and *δ* = 3). Therefore, our statistical data can be reliable, but still, underestimation of the results may be considered, and a larger prospective study should be conducted in the future to confirm our findings. Second, the effect of anticoagulation therapy was not assessed, as most AD patients with AF had already taken anticoagulation medicines. In other words, the results presented by patients with AD with AF may include the influence of these medications. Third, we only assessed WBC and CRP as inflammatory markers. Since chronic inflammation is expected to be one of the causes of WMLs, the relationship between inflammatory response and WMLs in dementia patients with AF should be comprehensively analyzed in the future.

## Conclusion

AF contributes to cognitive decline in patients with AD and amnestic MCI. PVWML may partially explain the involvement of AF in AD pathology. A larger prospective study is needed to confirm our findings in the future.

## Supplementary Information

**Additional file 1.**

**Additional file 2: Table S1.** Existence of CVD lesions.

**Additional file 3: Table S2.** Number of MBs.

**Additional file 4: Table S3.** The stepwise regression analysis of MMSE-J score with minimal BIC rule.

**Additional file 5: Table S4.** Stereotactic extraction estimation analysis for SPECT in AD and aMCI.

## Data Availability

The datasets used and analyzed during the current study are available from the corresponding author upon reasonable request.

## Abbreviations

AD: Alzheimer’s disease
AF: Atrial fibrillation
CMBs: Cerebral microbleeds
CRP: C-reactive protein
D: Deep
HR: Hazard ratio
IL-8: Interleukin-8
MCI: Mild cognitive impairment
MCP-1: Monocyte chemoattractant protein-1
MMSE-J: Japanese version of the Mini-Mental State Examination
MRA: Magnetic resonance angiography
NT-pro BNP: N-terminal pro-brain natriuretic peptide
PV: Periventricular
rCBF: Regional cerebral blood flow
SEE: Stereotactic extraction estimation
SPECT: Single-photon emission tomography
SR: Sinus rhythm
TNF-α: Tissue necrotizing factor-α
WBC: White blood cell
WML: White matter lesion
