# Supplementary figures and images for: Impact of atrial fibrillation on the cognitive decline in Alzheimer’s disease
Source: Alzheimers Res Ther. 2023 Jan 13;15:15. doi: 10.1186/s13195-023-01165-1 (PMC9838038; doi:10.1186/s13195-023-01165-1)

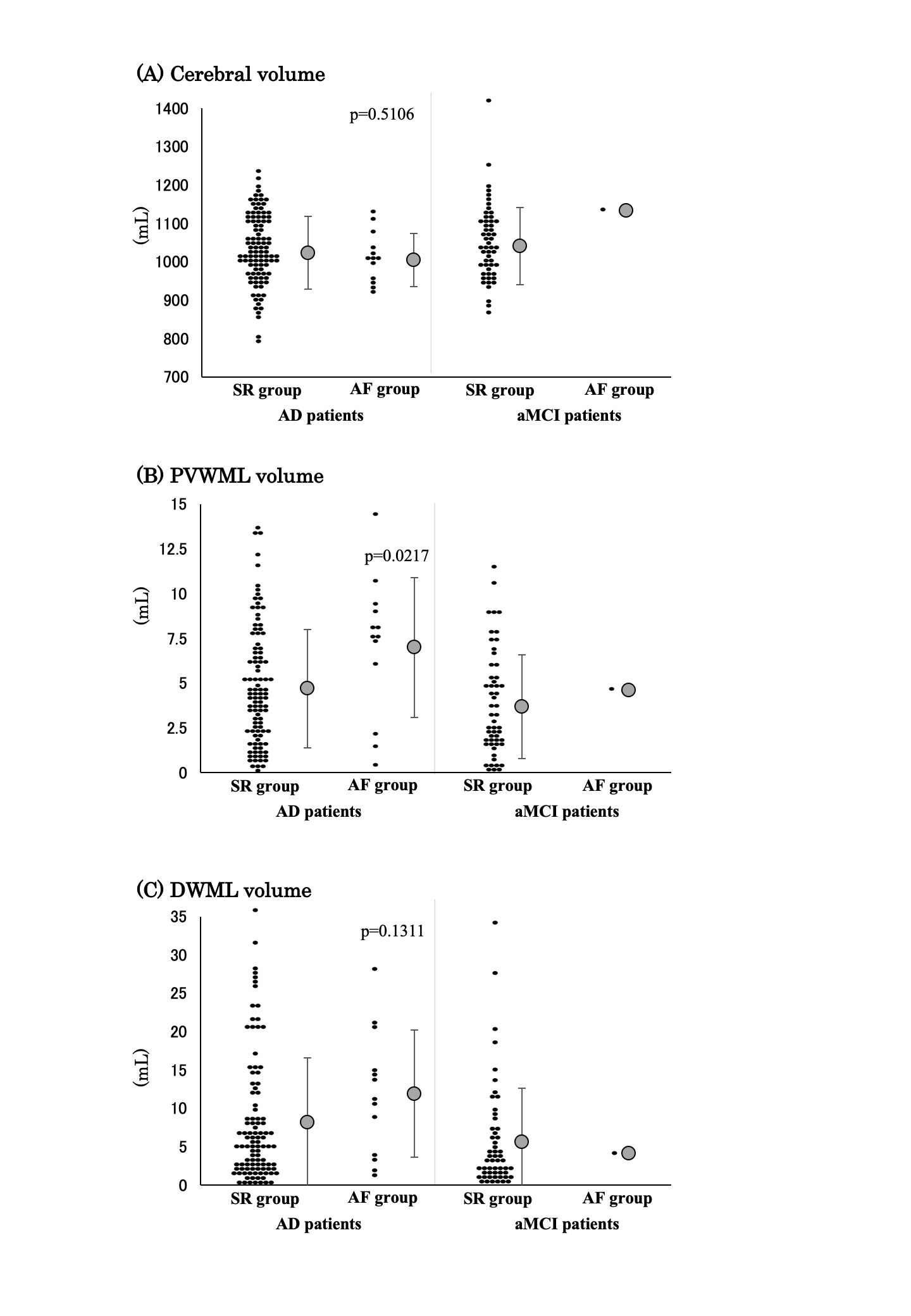

Supplement: Supplementary file 1 — Additional file 1. [file 13195_2023_1165_MOESM1_ESM.tiff]
