# Supplementary material for: Impact of atrial fibrillation on the cognitive decline in Alzheimer’s disease
Source: Alzheimers Res Ther. 2023 Jan 13;15:15. doi: 10.1186/s13195-023-01165-1 (PMC9838038; doi:10.1186/s13195-023-01165-1)
Supplement: Supplementary file 2 — Additional file 2: Table S1. Existence of CVD lesions. [file 13195_2023_1165_MOESM2_ESM.docx]

Supplemental Table 1. Existence of CVD lesions

|  | AF | SR | p |
| --- | --- | --- | --- |
| AD (n, %) | 2/13 (15.4%) | 9/102 (8.8%) | 0.6108 |
| aMCI (n, %) | 0/1 (0%) | 5/54 (9.3%) | - |

AF, atrial fibrillation; SR, sinus rhythm; AD, Alzheimer’s disease; aMCI, amnestic mild cognitive impairment.
