# Supplementary material for: Impact of atrial fibrillation on the cognitive decline in Alzheimer’s disease
Source: Alzheimers Res Ther. 2023 Jan 13;15:15. doi: 10.1186/s13195-023-01165-1 (PMC9838038; doi:10.1186/s13195-023-01165-1)
Supplement: Supplementary file 3 — Additional file 3: Table S2. Number of MBs. [file 13195_2023_1165_MOESM3_ESM.docx]

Supplemental Table 2. Number of MBs

|  | AF | SR | p |
| --- | --- | --- | --- |
| AD (ave.±SD) | 1.4±5.0 | 2.8±8.9 | 0.4800 |
| aMCI (ave.±SD) | 0 | 1.9±5.4 | - |

AF, atrial fibrillation; SR, sinus rhythm; AD, Alzheimer’s disease; aMCI, amnestic mild cognitive impairment.
