# Supplementary material for: Impact of atrial fibrillation on the cognitive decline in Alzheimer’s disease
Source: Alzheimers Res Ther. 2023 Jan 13;15:15. doi: 10.1186/s13195-023-01165-1 (PMC9838038; doi:10.1186/s13195-023-01165-1)
Supplement: Supplementary file 4 — Additional file 4: Table S3. The stepwise regression analysis of MMSE-J score with minimal BIC rule. [file 13195_2023_1165_MOESM4_ESM.docx]

Supplemental Table 3. The stepwise regression analysis of MMSE-J score with minimal BIC rule

|  | estimate value | p | R^2 |
| --- | --- | --- | --- |
| **Atrial fibrillation** | -0.9394 | 0.0347 | 0.0263 |
| **Diabetes mellitus** | -0.5595 | 0.1471 | 0.0385 |
| **CTR** | -0.0479 | 0.4163 | 0.0423 |
| **PVWML** | - | 0.8178 | 0.0426 |

CTR: cardiothoracic ratio, PVWML: periventricular white matter lesion.
