# Supplementary material for: Impact of atrial fibrillation on the cognitive decline in Alzheimer’s disease
Source: Alzheimers Res Ther. 2023 Jan 13;15:15. doi: 10.1186/s13195-023-01165-1 (PMC9838038; doi:10.1186/s13195-023-01165-1)
Supplement: Supplementary file 5 — Additional file 5: Table S4. Stereotactic extraction estimation analysis for SPECT in AD and aMCI. [file 13195_2023_1165_MOESM5_ESM.docx]

Supplemental Table 4. Stereotactic extraction estimation analysis for SPECT in AD and aMCI

|  | **AF** | **SR** | p |
| --- | --- | --- | --- |
| n | AD 11  aMCI 1 | AD 96  aMCI 51 |  |
| **SFG (L/R, ave.±SD)** | 71.3±26.5 / 73.9±29.2  74.8 / 62.7 | 65.5±26.7 / 64.3±28.7  61.2±29.4 / 59.6±28.9 | 0.4947 / 0.2974  na |
| **MFG (L/R, ave.±SD)** | 75.5±19.7 / 76.7±22.8  77.9 / 67.0 | 67.7±24.5 / 68.0±25.3  64.8±26.5 / 63.8±21.7 | 0.3152 / 0.2778  na |
| **Precuneus (L/R, ave.±SD)** | 71.4±34.0/ 73.6±34.8  69.5 / 87.1 | 67.6±31.0 / 70.4±31.6  63.7±31.8 / 64.2±31.8 | 0.7051 / 0.7532  na |
| **Thalamus (L/R, ave.±SD)** | 91.2±20.4 / 88.2±28.1  39.4 / 97.0 | 72.8±32.3 / 82.3±27.6  64.8±37.2 / 78.3±32.3 | 0.0676 / 0.4996  na |
| **PHG (L/R, ave.±SD)** | 81.8±17.2/ 86.5±16.1  85.7 / 69.4 | 76.8±28.8 / 78.0±26.2  67.4±31.3 / 67.9±33.3 | 0.5717 / 0.3002  na |
| **PCG (L/R, ave.±SD)** | 82.7±28.0 / 86.3±24.9  91.8 / 97.3 | 81.1±21.6 / 78.9±23.6  73.1±31.3 / 67.4±30.2 | 0.8254 / 0.3301  na |

AF, atrial fibrillation; SR, sinus rhythm; AD, Alzheimer’s disease; aMCI, amnestic mild cognitive impairment; SFG, Superior Frontal gyrus; MFG. Medial Frontal gyrus; PHG, Parahippocampal gyrus; PCG, Posterior Cingulate gyrus.
